# Avian influenza virus, *Streptococcus suis* serotype 2, severe acute respiratory syndrome-coronavirus and beyond: molecular epidemiology, ecology and the situation in China

**DOI:** 10.1098/rstb.2009.0093

**Published:** 2009-09-27

**Authors:** Ying Ma, Youjun Feng, Di Liu, George F. Gao

**Affiliations:** 1 CAS Key Laboratory of Pathogenic Microbiology and Immunology (CASPMI), Institute of Microbiology, Chinese Academy of Sciences (CAS), Beichen West Road, Beijing 100101, The People's Republic of China; 2 Beijing Institutes of Life Science, Chinese Academy of Science (CAS), Lincui East Road, Beijing 100101, The People's Republic of China

**Keywords:** avian influenza, *Streptococcus suis*, SARS-CoV, EHEC, PRRSV

## Abstract

The outbreak and spread of severe acute respiratory syndrome-associated coronavirus and the subsequent identification of its animal origin study have heightened the world's awareness of animal-borne or zoonotic pathogens. In addition to SARS, the highly pathogenic avian influenza virus (AIV), H5N1, and the lower pathogenicity H9N2 AIV have expanded their host ranges to infect human beings and other mammalian species as well as birds. Even the ‘well-known’ reservoir animals for influenza virus, migratory birds, became victims of the highly pathogenic H5N1 virus. Not only the viruses, but bacteria can also expand their host range: a new disease, streptococcal toxic shock syndrome, caused by human *Streptococcus suis* serotype 2 infection, has been observed in China with 52 human fatalities in two separate outbreaks (1998 and 2005, respectively). Additionally, enterohaemorrhagic *Escherichia coli* O157:H7 infection has increased worldwide with severe disease. Several outbreaks and sporadic isolations of this pathogen in China have made it an important target for disease control. A new highly pathogenic variant of porcine reproductive and respiratory syndrome virus (PRRSV) has been isolated in both China and Vietnam recently; although PRRSV is not a zoonotic human pathogen, its severe outbreaks have implications for food safety. All of these pathogens occur in Southeast Asia, including China, with severe consequences; therefore, we discuss the issues in this article by addressing the situation of the zoonotic threat in China.

## 1. Introduction and background

Infectious diseases remain a serious killer around the world, especially in developing countries. Man has not won the war on pathogens.

Since some cases of atypical pneumonia (later named as severe acute respiratory syndrome, SARS) were diagnosed in Guangdong Province of southern China, this ‘aetiology-unknown’ disease spread to Hong Kong and caused some fatal human cases at the end of 2002 and the beginning of 2003 (Drosten *et al.* 2003). Through great collaborative efforts worldwide SARS was quickly identified as a new disease caused by a new virus, SARS-associated coronavirus (SARS-CoV) (Ksiazek *et al.* 2003). The emergence of SARS, the rapid subsequent isolation of the aetiological agent and its rapid control have provided paradigms for such devastating pathogens in modern society through effective control using modern technologies, including genomics (Feng & Gao 2007).

SARS-CoV is a very good example of an infectious pathogen in the modern world spreading quickly to many parts of the globe owing to extensive mobility of modern humans. Discovery of the animal origin of the SARS-CoV identified this agent as a zoonotic pathogen (Guan *et al.* 2003). On the one hand, we enjoy life with handy high technology, including convenient travel, but on the other hand, we face persistent challenges from nature, including animal-borne diseases, such as SARS. Threats from zoonotic or animal-borne pathogens are seriously increasing. Interspecies transmission of highly pathogenic H5N1 avian influenza virus (AIV), especially with the ever-increasing number of human cases (Gambotto *et al.* 2008), made zoonotic diseases more relevant than ever to human health. Most newly identified pathogens are animal-borne or zoonotic and many arose from Asia, especially Southeast Asia, including China, making the situation in this region critically important for disease control and surveillance around the world. Southeast Asia is considered as an epicentre of some important emerging infectious diseases (Shortridge & Stuart-Harris 1982). Therefore, we discuss in this article the epidemiology, distribution, molecular pathogenesis and control measures for SARS-CoV, AIV, *Streptococcus suis* serotype 2 (SS2), enterohaemorrhagic *Escherichia coli* and porcine reproductive and respiratory syndrome virus (PRRSV), addressing the situation in China.

## 2. Avian influenza virus: H5N1 and H9N2

Although highly pathogenic avian influenza (HPAI) virus has been a known pathogen of domestic chickens for over 100 years, its importance and awareness to the public has arisen after human infections with H5N1 HPAI virus were reported. Since the first fatal human case occurred in May 1997 in Hong Kong SAR (Special Administrative Region) (Claas *et al.* 1998), H5N1 has spread to cause infection and death in many species including domestic birds, waterfowl and mammals (i.e. tigers, dogs, cats and human beings). Surprisingly, the virus was able to cause lethal infection in wild waterfowl, the natural reservoir host of AIVs. Like other influenza A viruses, HPAI virus H5N1 has eight gene segments, encoding 11 proteins, except that in some lineages the *PB1-F2* gene is truncated (Zell *et al.* 2007). Among the 11 proteins, haemagglutinin (HA) and neuraminidase (NA) are the surface antigens and are responsible for virus attachment to cells and entry and release from infected cells, respectively. There are two major ways by which a virus may alter virulence and host range. One is gene reassortment, caused by gene segment exchange between virus strains, and the other is antigenic drift, which is due to the accumulated changes of amino acids under pressure from the immune system or other factors. From the so-called ancestor strain A/Goose/Guangdong/1/1996 (H5N1), the current circulating H5N1 viruses have experienced diverse gene reassortment events with heterogeneous gene segments deriving from viruses of wild birds (of known or unknown subtypes) or from co-circulating H5N1 viruses, resulting in the emergence of over 20 different genotypes.

**Figure 1.**
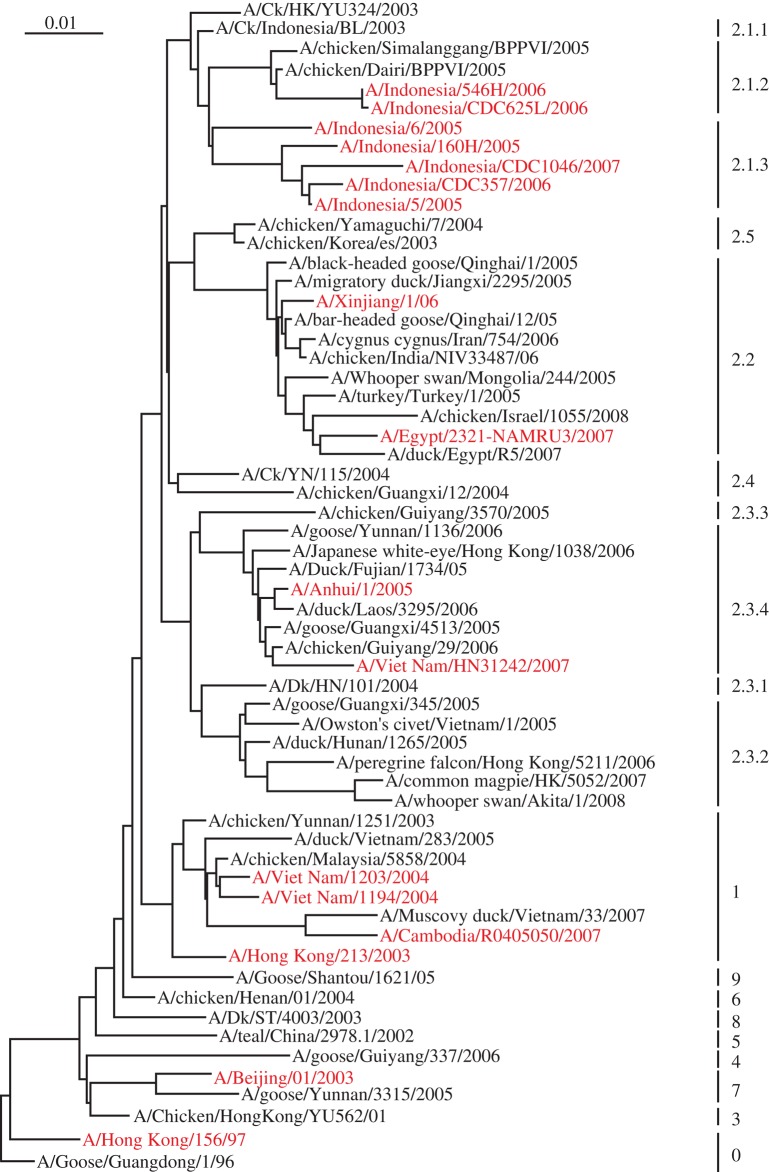
Phylogenetics of the H5N1 HA of the currently circulating strains. The representative viruses' HA sequences were downloaded from NCBI influenza virus resource (Bao *et al.* 2008). The classification is according to WHO's definition and human-infected viruses are labelled in red.

Within currently circulating H5N1 viruses, gene reassortment is recognized only in their internal genes, while the surface glycoproteins (HA and NA) are evolving under pressure from the host immune system or other systems. As far as HA is concerned, there are 10 main clades classified according to the nucleotide sequences, and one of the most prevalent clades, Clade 2, has been further divided into five subclades and seven third-order clades (figure 1). Among those clades, Clade 0 was prevalent in southern China, including Guangdong Province and Hong Kong SAR, and some sporadic cases in the nearby area, such as Fujian Province and Shanghai Autonomous City. Viruses in Clade 0 comprise the early-isolated strains in Hong Kong and mainland China, and some of them were the cause of the outbreak in Hong Kong in 1997. Clade 1 viruses have been isolated mainly in Vietnam and Thailand and also found circulating in Cambodia, Malaysia and southern China. Viruses of this clade were responsible for the outbreaks in Vietnam and Thailand in 2004, which resulted in many human deaths.

Clade 2 exhibits the most complicated phylogeny. Viruses from Clade 2.1 are mostly isolated from Indonesia, with 2.1.1 and 2.1.3 from eastern Indonesia and 2.1.2 from western Indonesia (Smith *et al.* 2006*b*). Virus with the most similarity to Clade 2.1 was isolated from chickens in Hong Kong, and further surveillance and analyses showed that it might have come from Hunan Province, China, probably via poultry trade (Wang *et al.* 2008*c*). Clade 2.2 viruses are well known as the Qinghai Lake-related (QHR) viruses, and will be discussed subsequently. Clade 2.3 viruses consist of four groups: viruses in Clade 2.3.1 are mainly from domestic birds of Hunan Province; viruses in Clade 2.3.2 are from Yunnan, Guangxi and Guangdong Provinces of China and from Vietnam; most Clade 2.3.3 viruses are from Guizhou Province of southern China and Clade 2.3.4 viruses are known as Fujian-Like (Smith *et al.* 2006*a*). Clade 2.3.4 viruses are a mixed group as they are isolated from different provinces of southern China, such as Guangdong, Guangxi, Guizhou, Yunnan and Hong Kong and the nearby country, Laos. Clade 2.3.4 includes some viruses isolated from human cases, such as H5N1 infection in a pregnant woman in Anhui Province (Shu *et al.* 2006). Viruses from Clades 2.4 and 2.5 were from some sporadic cases mainly in the east and central parts of Mainland China and in Japan and Korea (isolates 2003–2004). The virus source for the outbreaks in Japan and Korea is uncertain; some blame duck meat transportation (Lee *et al.* 2005; Mase *et al.* 2005), while others postulate that migratory birds played a role (Kilpatrick *et al.* 2006). The majority of viruses in Clades 3–9 were isolated in southern and southeastern China, such as Fujian, Guangdong, Guangxi, Guizhou, Yunnan and Hong Kong.

**Figure 2.**
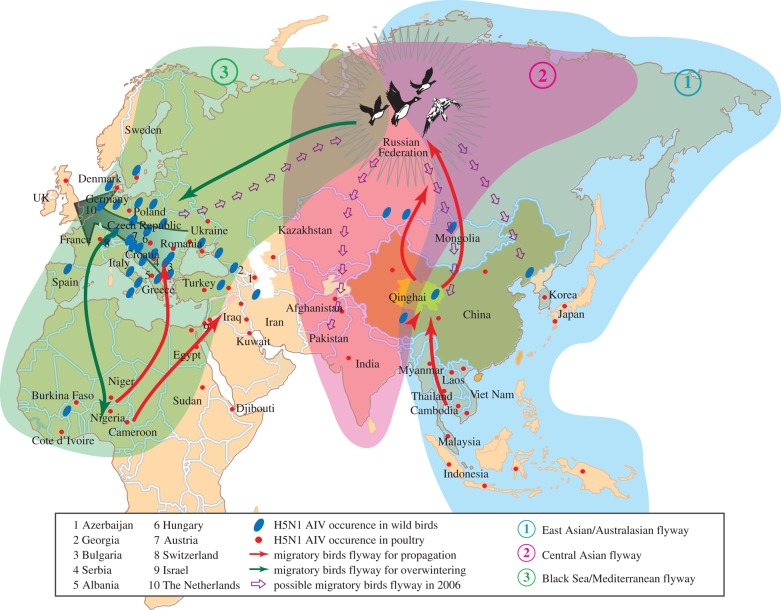
Adapted from Wang *et al.* with permission. The potential routes of migratory birds from 2005 to 2006 and the occurrence of H5N1 are shown. Three flyways are shaded in green, blue and pink. The red and blue dots illustrate the H5N1 occurrence. The arrows in red, green and purple denote the directions of the migratory birds. The coloured shadows are the ranges of different flyways. The grey arrow in Europe represents the cold weather. The cartoon birds highlight the congregation area of the wild birds from three different flyways.

Among all known H5N1 viruses, those in Clade 2.2 appear to be the most widespread. Since the outbreak occurred in migratory waterfowl around Qinghai Lake in 2005 (Chen *et al.* 2005; Liu *et al.* 2005), Clade 2.2 virus has spread to Mongolia, Russia, the Middle East, Europe and Africa and has resulted in fatal human infections in China, the Middle East and Africa. Clade 2.2 virus is highly pathogenic to wild waterfowl and has killed nearly 10 000 birds since 2005 (Chen *et al.* 2005, 2006; Liu *et al.* 2005; Wang *et al.* 2008*a*). The widespread occurrence begs the question of how did this kind of HPAI virus spread worldwide? It has been proposed that migratory birds and/or wild bird trade played a key role (Kilpatrick *et al.* 2006; Wang *et al.* 2008*a*). From the analyses of the re-emergence of H5N1 virus in Qinghai Lake in 2006, our group extrapolated the potential routes, through which the QHR H5N1 virus is circulating around the world (figure 2) (Wang *et al.* 2008*a*). According to this hypothesis, the migratory birds are proposed to carry viruses from Qinghai Lake north to Mongolia and Siberia where birds from different migratory flyways are gathered for breeding (congregation area). Some birds flying to the southwest are proposed to have become infected and brought the viruses to the Mediterranean for wintering. In this way, viruses were transferred to Europe and caused outbreaks in Romania, Turkey, Croatia and Ukraine in the late autumn of 2005. By aid of the waterfowl movements to the north and west in January, the Clade 2.2 virus spread through many European countries (Kilpatrick *et al.* 2006). The Mediterranean is another gathering site for migratory birds. We hypothesized that birds flying south from here carried viruses into Africa, while some carried them back to Siberia, resulting in spread to Central and Southern Asia, and finally back to Qinghai Lake the following spring. We propose that there are mainly three migratory bird flyways involved in the Clade 2.2 virus circulation (figure 2), that is: the Eastern Asian/Australasian flyway responsible for virus spread from Qinghai Lake to Siberia and to Korea, Japan and southeastern Asia; the Black Sea/Mediterranean flyway responsible for virus spread from Siberia to Europe and Africa; and the Central Asian flyway for virus spread to Central Asia and then back to Qinghai Lake (Wang *et al.* 2008*a*).

Migratory birds as carriers have resulted in Clade 2.2 strains spreading worldwide (visualized by Declan Butler's time-enabled map for H5N1 spread, http://www.nature.com/avianflu/google-earth/index.html), so the surveillance of the migratory birds is also important. Surveillance of Qinghai Lake shows that the number of infected birds decreased in 2007 and dramatically reduced in 2008 (L. Li, X. Hu, D. Liu & G. F. Gao 2009, unpublished data). He and colleagues have deposited in Genbank a Qinghai Lake 2008 virus A/environment/Qinghai/1/2008 (H5N1), which was isolated from the faecal sample. It should be noted that this strain did not fall within Clade 2.2 but Clade 0. The question of how the bird population was able to recover from the outbreak needs to be addressed. Is it because of virus adaptation to hosts or does the reduced population size limit the virus circulation? Further analyses need to be applied to answer these questions.

Viruses responsible for human cases of infection have come from Clades 0, 1, 2.1.2, 2.1.3, 2.2, 2.3, 4 and 7 (figure 1). In total, the cumulative number of human cases reached 405 with 254 fatalities (see http://www.who.int/csr/disease/avian_influenza/country/cases_table_2009_02_05/en/index.html). Among them, seven human infections with four deaths have occurred in China during early 2009. Clade 1 viruses are responsible for the most human cases in Vietnam, and Clade 2.1 viruses in Indonesia; these account for more than half of the human cases worldwide. Between 2005 and 2008, there were sporadic human cases in China, most of which were caused by Clade 2.3.4 viruses. These infected people were reported to have handled live poultry and there is no proof of human-to-human infection, though there is one suspected son–father case (Wang *et al.* 2008*b*). There was another human case in China caused by virus (A/Xinjiang/1/06) in Clade 2.2. H9N2 causes a milder form of avian influenza; however, co-infection with *Staphylococcus* spp. or *Haemophilus* spp. increases its virulence to chickens (Kishida *et al.* 2004) and probably is the key reason for lethality (Li *et al.* 2005*b*). Infection by H9N2 was first detected in turkeys in Wisconsin in 1966 (Homme & Easterday 1970), then H9N2 subtype viruses were found circulating in shore birds and wild ducks in North America (Kawaoka *et al.* 1988). In Asia, infections by the H9 subtype of AIV were reported in chickens and pigs since the late 1990s (Peiris *et al.* 1999*a*; Guo *et al.* 2000), and in 1999 the first confirmed cases of human infection with H9N2 virus shocked the world (Peiris *et al.* 1999*b*). Recently a Hong Kong H9N2 virus isolated in 1997 was deposited in GenBank (A/Hong Kong/W213/1997). Two human cases were associated with this outbreak and both the 13-month-old and the 4-year-old girls who were infected had uncomplicated influenza-like illness and fully recovered after medical treatment. Further studies on those who had contact with those young patients showed no evidence of human-to-human transmission (Uyeki *et al.* 2002). There have also been reports of H9-like illness in Guangdong Province, China, in 1998 and later (Guo *et al.* 1999). In total, there are only a few cases of human infections of H9N2 and none proved fatal.

**Figure 3.**
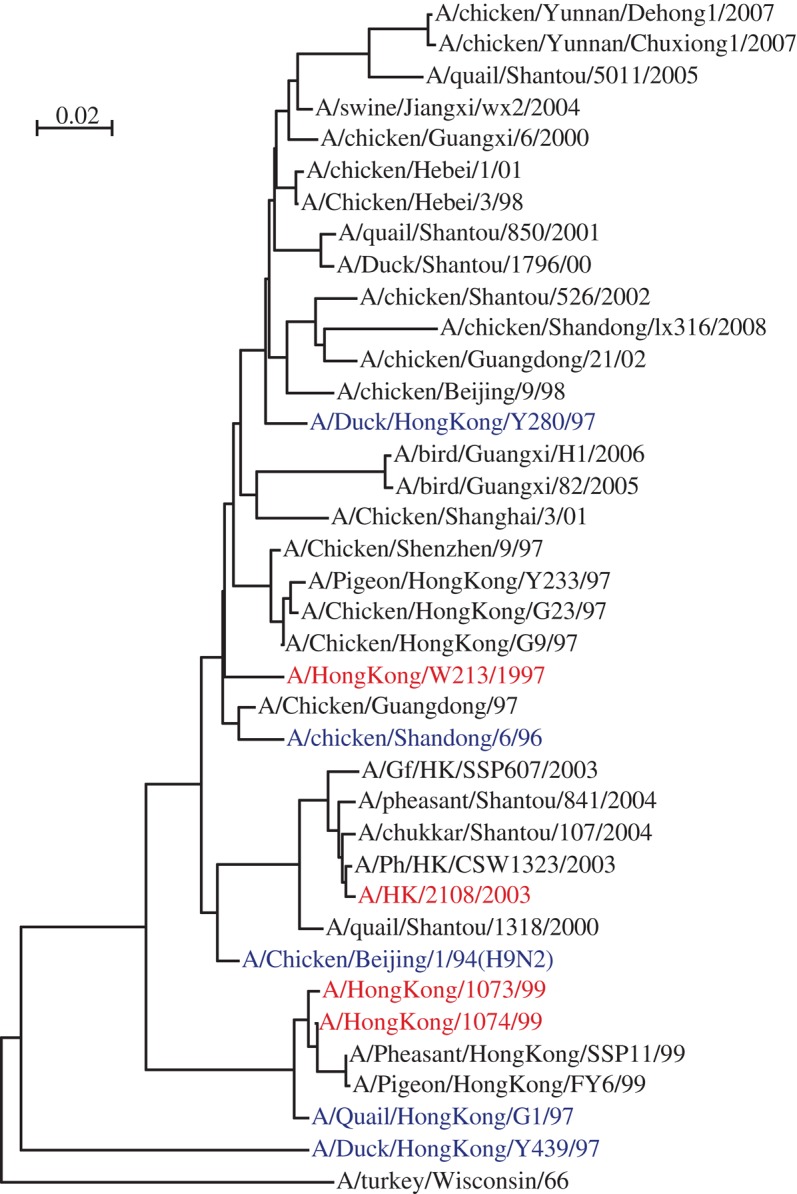
Phylogenetics of H9N2 HA. The tree was rooted with A/turkey/Wisconsin/66 (H9N2). The human strains are in red, and those strains mentioned in the text are in blue.

In mainland China, the first H9N2 virus was isolated from chickens in Guangdong Province in 1994 (Chen *et al.* 1994; Li *et al.* 2003) and, since 1998, an inactivated vaccine derived from A/chicken/Shandong/6/1996 (H9N2) has been used widely in chicken farms, China (Li *et al.* 2005*b*). From surveillance and phylogenetics analysis, the HA genes of almost all H9N2 isolates from poultry of China are believed to evolve from that of the strain A/chicken/Beijing/1/1994 (H9N2) (Li *et al.* 2005*b*) (figure 3). In Hong Kong SAR, the H9N2 isolates from avian species and live bird markets are mainly grouped into three groups, represented by A/Quail/Hong Kong/G1/1997 (H9N2) (G1), A/Duck/Hong Kong/Y280/1997 (H9N2) (Y280) and A/Duck/Hong Kong/Y439/1997 (H9N2) (Y439) (Guan *et al.* 1999). The two 1999 human isolates in Hong Kong are G1-like viruses (figure 3). Interestingly, the G1-like strains have internal genes similar to those of the H5N1 viruses that caused 18 cases of human infections in Hong Kong in 1997 (Lin *et al.* 2000). Neither the origin of those shared genes nor their effectiveness in the inter-species transmission is known and this should be addressed in future studies.

## 3. *Streptococcus suis* serotype 2

*Streptococcus suis* has been known as a swine pathogen for a long time and the first human case was reported in 1968 in Denmark (Dupas *et al.* 1992). Since then, over 400 human cases, mainly presenting with meningitis, arthritis or other milder symptoms, have been recorded, covering nearly 30 countries (e.g. Thailand, China, Canada, etc.). The causative agent, *S. suis*, is a Gram-positive, facultatively anaerobic *coccus* of which 35 serotypes have been proposed based on differential expression of capsule antigens. Among 35 serotypes, SS2 is a prevalent virulent strain, frequently isolated from pigs, especially in affected piglets, and has developed into a serious zoonotic entity, posing a great challenge to global public health. Effective prevention and therapeutic treatments are not available thus far, which is, in part, due to lack of comprehensive understanding of the molecular pathogenesis underlying this enigmatic bacterium.

In 1998, a severe form of human SS2 infection, streptococcal toxic shock syndrome (STSS), was identified, in Jiangsu Province, China, resulting in 14 human deaths (Tang *et al.* 2004). Again, in 2005, an STSS human outbreak caused by SS2 was found in Sichuan Province, China, with 38 human deaths and over 200 human infections (Tang *et al.* 2006; Yu *et al.* 2006). In this outbreak, more than 600 pigs in the epidemic area were found to be severely infected by SS2. In 2008, a case of human SS2 infection with manifestations of STSS was reported in Australia, which was associated with occupational exposure to SS2 from animal carcasses (Tramontana *et al.* 2008), implying the spread of the severe from of SS2 across Southeast Asia and the Pacific area.

Initially, STSS was caused mainly by *Staphylococcus aureas* (Todd *et al.* 1978). The term STSS was later used to describe severe human-invasive infection caused by Group A streptococci, *Streptococcus pyogenes* (http://www.cdc.gov/epo/dphsi/casedef/streptococcalcurrent.htm; Cone *et al.* 1987). To be more cautious, Xu and colleagues (Zheng *et al.* 2008) referred to SS2 infection with STSS symptoms in humans as streptococcal toxic shock-like syndrome. From our collaborative efforts we have used the term STSS for severe invasive infection of SS2 in humans observed in 1998 and 2005 outbreaks (Tang *et al.* 2006).

STSS is defined by certain clinical criteria: (i) sudden onset of high fever; (ii) blood spots and petechia; (iii) clear erythematous blanching rash; (iv) hypotension diarrhoea; and (v) dysfunction of multiple organs, such as acute respiratory distress syndrome, liver and heart failure, disseminated intravascular coagulation and acute renal failure (Tang *et al.* 2006). In addition to STSS, meningitis was also observed in the two Chinese outbreaks (1998 and 2005). Epidemiological surveys have indicated that nearly all the patients show a history of close contact with diseased pigs and pork-derived products. Backyard cultivation of pigs, a widespread agricultural practice in many Asian countries, is considered a primary risk factor, as well as informal slaughtering and consumption at home, and even the illegal commercialization of diseased pigs (Yu *et al.* 2006). Nevertheless, there can be no doubt that the emergence of highly virulent SS2 strains plays a key role in severe SS2 outbreaks in China. This proposal was validated by subsequent investigations of these pathogens at molecular and genomic level (Chen *et al.* 2007). Indeed, extensive analyses have demonstrated that the Chinese SS2 epidemic strains could be a variant that is distinct from P1/7, an international representative strain of SS2 (http://www.sanger.ac.uk/Projects/S.suis; Chen *et al.* 2007). Last year, in collaboration with China Shenzhen CDC, we isolated three more SS2 strains from meningitis patients in Shenzhen and Chengdu Cities, China, and confirmed their distinct characteristics (Feng *et al.* in press). Based on sequence typing (ST), Xu and colleagues suggested that ST7 is prevalent in China (Ye *et al.* 2006) but this cannot explain the distinct characteristics of the STSS disease in two severe outbreaks in China as ST7 can be found in other countries (Tramontana *et al.* 2008). Further molecular analysis of the bacterial strains, including genome sequencing, structural biology and proteomics, should aid our understanding of this emerging zoonotic infection. Two Chinese STSS associated strains of SS2 were selected for whole-genome sequencing (based on a preliminary restriction fragment length polymorphism study), which showed that the two Chinese STSS strains are of the same genotype as each other but different from the international standard strain (Chen *et al.* 2007). One of the Chinese strains is 98HAH12, isolated from an STSS patient that died in the 1998 SS2 outbreak, and the other is 05ZYH33, a human isolate from a diseased STSS patient in the 2005 epidemic (Tang *et al.* 2006). Comparative genomics revealed that a DNA fragment of about 89 kb, designated as 89K, is present in the two Chinese strains (98HAH12 and 05ZYH33), while absent in the P1/7 strain (http://www.sanger.ac.uk/Projects/S.suis) (Chen *et al.* 2007). This 89K fragment is proposed to be a putative pathogenicity island (PAI) based on the following criteria (Chen *et al.* 2007): (i) its mosaic architecture includes homologous genes found in different bacteria including *Enterococcus faecalis*; (ii) its GC content (36.8%) is much lower than that of the core genome (41.1%); (iii) its size, 89K, falls into the length range of a PAI (10–200 kb); (iv) it is adjacent to the 39-terminus of the 50S ribosome gene, a house-keeping gene, similar to the tRNA gene adjacent to PAIs in Gram-negative bacteria; (v) it has two mobile genetic element; (vi) it is only present in the two virulent strains, 98HAH12 and 05ZYH33, and not in an avirulent isolate, 05HAS687; a series of virulence-associated factors are mapped to this region, including a zeta-toxin, three ABC transporter cassettes controlling efflux and influx across cell membranes for small substances and two two-component signal transduction systems (TCSTS or TCS).

Genetic studies indicate that disruption of the SalK/SalR TCS inside the 89K (Li *et al.* 2008), attenuates greatly the high virulence of this pathogen (strain O5ZYH33 was used), whereas functional complementation restores virulence in experimental infection of piglets. The attenuated virulence of the mutant △*SalK/SalR* can be, in part, attributed to decreased colonization capability in susceptible tissues of piglets and lower resistance to polymorphonuclear leukcocyte-mediated killing.

In a different approach, Xu and colleagues (Ye *et al.* 2008) recently proposed a two-stage hypothesis to explain STSS in the Chinese outbreaks. They used the SC84 SS2 strain (isolated from a human STSS patient in the 2005 Sichuan outbreak) and found that this ST7 bacterium possesses a stronger capacity to stimulate T cells, naive T cells and peripheral blood mononuclear cell proliferation than does an ST1 strain. Therefore, the pathogenesis of the STSS-causing SS2 involves two stages: in the first stage, the infected patients experience a kind of ‘immunological over-reaction’, such as the ‘cytokine storm’ in human SARS or AIV infections. This reflects the STSS manifestation of the SS2 human infection. In the second stage, if the patients survive the over-reaction or the patients do not experience this stage, the patients' immunological responses are in a more controlled manner, and they might experience a mild disease, such as meningitis or arthritis. This hypothesis needs further studies in the future.

## 4. Severe acute respiratory syndrome-associated coronavirus

In the winter of 2002, an unknown atypical pneumonia disease was observed in Guangdong Province, China, and the disease was soon seen in Hong Kong SAR, China (Drosten *et al.* 2003). This ‘atypical pneumonia’ disease, later named as SARS, was a newly emerging viral disease, which caused panic worldwide, owing to the lack of awareness and knowledge for hospital infection control (Peiris *et al.* 2003; Christian *et al.* 2004). In total, more than 8400 SARS patients were recorded, in which over 800 people died (Christian *et al.* 2004; Feng & Gao 2007). Faced with this unparalleled SARS epidemic, an international collaboration was instigated towards elucidating the agent underlying this mysterious pandemic. A novel type of coronavirus, referred to as SARS-CoV, was identified to be the aetiological agent soon after the outbreak (Ksiazek *et al.* 2003). Scientific communities worldwide regarded it as a viral paradigm of an emerging infectious disease (Thiel *et al.* 2003; Berger *et al.* 2004; Osterhaus *et al.* 2004) and conducted extensive studies including molecular epidemiology (Zhao 2007), virology/immunology/vaccine development (Chen & Subbarao 2007; Satija & Lal 2007; Frieman *et al.* 2008; Guo *et al.* 2008) and structural proteomics (Bartlam *et al.* 2007) with the aim of searching for therapeutics and treatments for this serious infectious disease. As we wrote this review, the SARS-related literature has strikingly accumulated over 5255 publications in PubMed during the limited period of 5 years since its description and discovery.

The global spread of SARS has been attributed to an individual who was initially infected by atypical pneumonia in Guangdong Province (Poon *et al.* 2004). This patient, later referred to a ‘super-spreader’, travelled to Hong Kong SAR prior to, it has been suggested, his succumbing to this disease (Chim *et al.* 2003; Guan *et al.* 2003, 2004). During this period, he unwittingly infected a few persons who in turn transmitted this pathogen to many different countries in the world including Canada, Europe, Singapore, etc. through air travel (Chim *et al.* 2003; Guan *et al.* 2003, 2004). As clinical observations accumulated, WHO was able to term this new type of disease as SARS and proposed clinically diagnostic criteria for the SARS patient as follows: (i) close contact with a known SARS patient or having infected other people; (ii) high fever (greater than 38°C) and symptoms of respiratory illness; (iii) leukocyte count less than 10.0 × 10^9^ l^−1^; (iv) radiographic evidence for pneumonia-like infiltrates or respiratory distress syndrome on chest X-ray; and (v) failure in antimicrobial treatments (within 72 h) (Poon *et al.* 2004; Feng & Gao 2007). Most of those patients, if not all, exhibited a typical incubation period that varied from 2 to 10 days (Berger *et al.* 2004; Poon *et al.* 2004), and the overall death rate was about 10 per cent during these SARS outbreaks (Berger *et al.* 2004; Stockman *et al.* 2006). Among them, mainland China was the most severely affected region, with over 5000 cases reported (Peiris *et al.* 2004).

The aetiological pathogen for the atypical pneumonia, on the basis of Koch's postulates, was isolated and confirmed to be a novel member of group II coronaviruses, SARS-CoV (Drosten *et al.* 2003; Ksiazek *et al.* 2003). Within 10 days, two different research groups reported the whole genomes of two SARS-related viruses, pinpointing that SARS-CoV possesses a single strand, positive sense (+) RNA genome of approximately 29.7 kb, harbouring 14 functional open reading frames that can be processed into four structural proteins, eight accessory proteins and 16 non-structural proteins (Marra *et al.* 2003; Rota *et al.* 2003). It is commonly accepted that SARS-CoV emerged as an infectious entity and evolved a capability to overcome interspecies barriers to infection and causes serious disease, resulting in worldwide public health concerns and in some areas a reaction close to panic. However, it remains obscure what circumstances disrupted the natural ecological balance of the virus, allowing it to extend from a natural reservoir and adapt to human hosts (Li *et al.* 2005*c*, 2006; Wang & Eaton 2007).

Since the SARS outbreaks, Chinese scientists have made much progress on our understanding of SARS/SARS-CoV (Bartlam *et al.* 2007; Wang & Eaton 2007). Initially, an important clue was obtained from a field epidemiological survey of live-animal markets in Guangdong Province, China, where it was observed that 13–40% of wild animal traders and slaughterers were sero-positive for the SARS-CoV (Poon *et al.* 2004; Shi & Hu 2008). This directly led to a provocative proposal that SARS may have emerged from an unknown animal reservoir (Poon *et al.* 2004; Shi & Hu 2008). Subsequently, SARS-like viruses were isolated from Himalayan palm civets (*Paguma larvata*) and a raccoon dog (*Nyctereutes procyonoides*) at an animal market in Shenzhen city, China; these virus genome sequences exhibited 99 per cent similarity to SARS-CoV (Guan *et al.* 2003). As a result of these observations, large-scale culling of civets was carried out in southern China but contrary to expectation, further field investigations and experimental evidence showed that civets may not be the natural reservoir for SARS-CoV, but rather they are susceptible animal hosts (Kan *et al.* 2005). Thus, the search for the origins of SARS-CoV was shifted quickly to other animal candidates. In 2005, a group led by Prof. Zhang at the Institute of Zoology, Chinese Academy of Sciences, announced the exciting discovery that bats serve as natural reservoirs for SARS-like coronaviruses (Li *et al.* 2005*c*). It has been recognized for a long time that bats are natural hosts for many zoonotic agents including Hendra and Nipah viruses (Enserink 2000; Halpin *et al.* 2000). Increasing demand of bats and/or bat products in food and traditional medicine markets have existed in certain Asian countries, notably in southern China. Thereby, it is rational that SARS-like viruses may jump to humans from bats, adapt themselves to overcome the interspecies barrier and finally result in human-to-human transmission as in the SARS pandemic (Li *et al.* 2005*c*; Wang & Eaton 2007; Shi & Hu 2008).

From the analysis of the molecular epidemiology/evolution of SARS-CoV isolates, Zhao's group conducted intensive comparative genomic studies involving 61 isolates of SARS-CoVs sampled from the early, middle and late phases of the SARS outbreak as well as two viral genomes from palm civets. This excellent work provided key clues to the evolution of SARS CoVs and supported the animal origins of the human SARS epidemic (Chinese SARS Molecular Epidemiology Consortium, 2004; Kan *et al.* 2005). Follow-up studies in Zhao' group further delineated molecular insights into cross-host evolution of SARS-CoV in palm civets and humans (Song *et al.* 2005). In addition, Xu *et al.* (2004*a*) introduced the concept of quasi-species into the newly emerging virus, SARS-CoV, a concept well recognized in other serious human pathogens such as hepatitis C virus (Quesnel-Vallieres *et al.* 2008) and human immunodeficiency virus (HIV; Salemi *et al.* 2007). This concept suggested that genetic variants of SARS-CoV form a pool of heterogeneous viruses in individual patients, mainly due to poor fidelity of its RNA polymerase (Xu *et al.* 2004*a*).

Research concerning SARS-related prevention/therapeutics has produced a series of exciting results. Inactivated SARS vaccine elicits potent spike protein-specific neutralizing antibodies that block receptor binding and virus entry (He *et al.* 2004). Similarly, a DNA vaccine encoding S glycoprotein can induce production of neutralizing antibody, as well as protective immunity in a mouse model (Wang *et al.* 2005). Transgenic plants (e.g. tomato and tobacco) have been shown to successfully produce S1 antigen of SARS-CoV (Pogrebnyak *et al.* 2005). More excitingly, Zhong's group evaluated small interfering (si)RNA inhibitors of SARS for efficacy and safety in a rhesus macaque (*Macaca mulatta*) SARS model, indicating that siRNA-based SARS-CoV inhibitors can serve as useful therapeutic agents (Li *et al.* 2005*a*). The combination of two non-competing human monoclonal antibodies CR3014 and CR3022 have been shown to have a good potential to control immune escape (ter Meulen *et al.* 2006). Based on the mechanism of type I membrane fusion employed by SARS-CoV (Zhu *et al.* 2004), our group and others reported the crystal structure of SARS-CoV membrane fusion core (Xu *et al.* 2004*b*) and developed some recombinant protein inhibitors targeting virus fusion and entry (e.g. HR2, HR121 and HR212 in our group), exhibiting high stability and potent inhibitory activity on entry of the HIV/SARS pseudoviruses (Ni *et al.* 2005). Similarly, Rao's group have reported on 3CL structure-based wide-spectrum inhibitors targeting *Coronavirus* with SARS-CoV included (Yang *et al.* 2005). Particularly, two kinds of Chinese herbal medicine-derived small molecules (tetra-*O*-galloyl-beta-d-glucose and luteolin) were demonstrated to share potent anti-SARS-CoV activities using a wild-type SARS-CoV infection system (Yi *et al.* 2004). SARS really represents a very good example of an infectious agent in a modern society, emerging suddenly and devastatingly, but conquered extremely quickly.

## 5. Enterohaemorrhagic *E. coli* O157:H7

*Escherichia coli* is found in normal intestine flora in both humans and animals (Cooke & Ewins 1975). Some serotypes of *E. coli* can cause disease ranging from mild forms to fatal cases and it is usually food-borne. Enterhaemorrhagic *E. coli* (EHEC) causes diarrhoea, haemorrhagic colitis, haemolytic uraemic syndrome (HUS), thrombotic thrombocytopenic purpura, etc. (Mead *et al.* 1999). Under the name of EHEC, there are several *E. coli* serotypes/groups causing disease but the most severe and prevalent is O157:H7. The first EHEC O157:H7 outbreak caused by food poisoning was reported in the USA in 1982 (Riley *et al.* 1983) and outbreaks have subsequently been found all over the world, with several hundred severe outbreaks worldwide and mortality reaching 5–10% (Hedden 2008). In 1986, the first cases of an EHEC O157:H7 outbreak in China were found in Xuzhou city, Jiangsu Province (Xu & Qi 1996). Later, between 1999 and 2000, several outbreaks in the middle-eastern areas of China, including Xuzhou city, were reported and this represents the most severe outbreak in the world, lasting a long time with high mortality.

**Figure 4.**
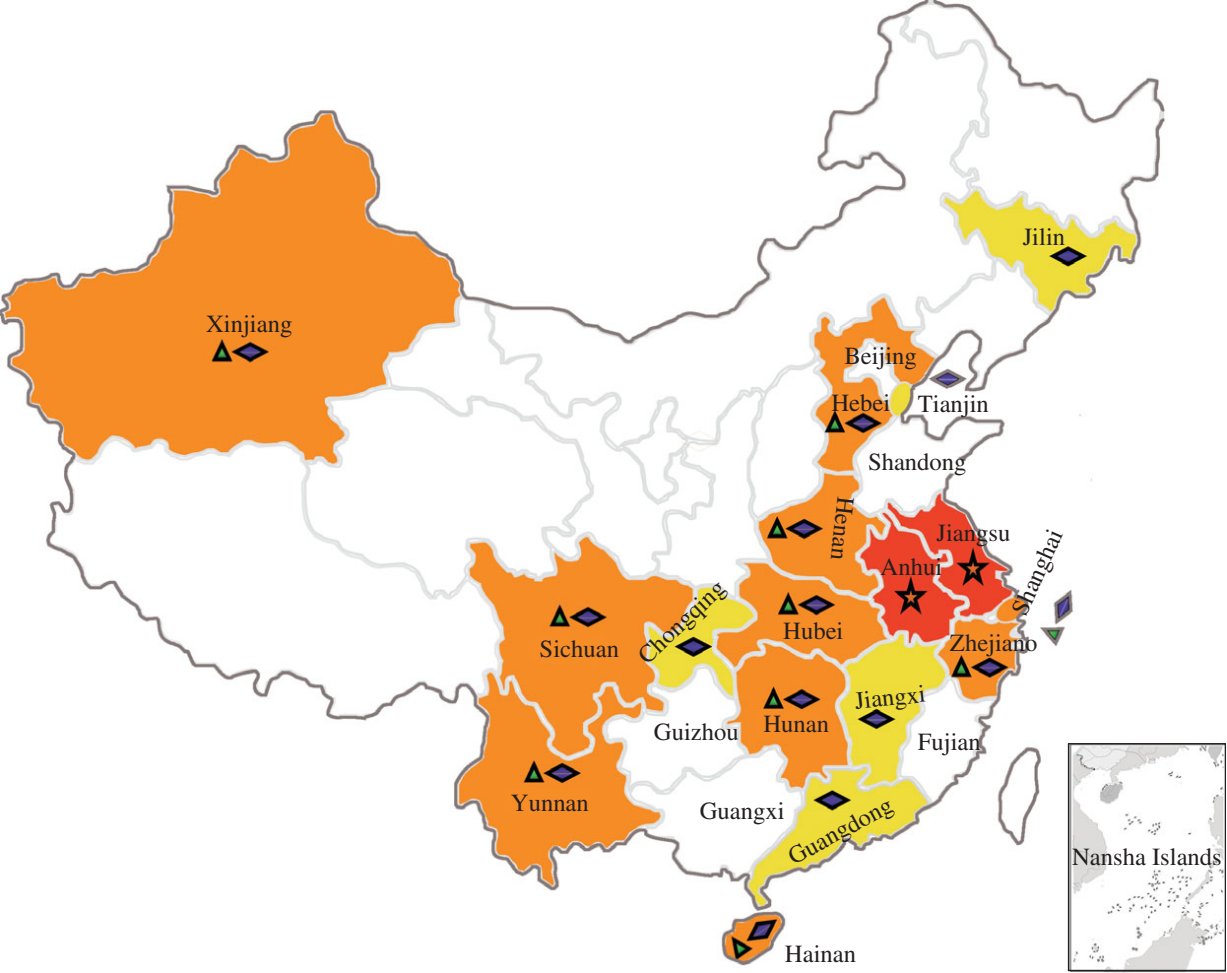
Map of China with EHEC outbreaks and isolations from animals. Stars represent the large-scale outbreak in 1999 in Eastern provinces, Anhui and Jiangsu. Triangles represent human infections. Diamonds represent animal isolation.

Since then, there have been several more outbreaks in China and the causative agent O157:H7 *E. coli* has been isolated in half of the Chinese territory. The outbreaks and isolations are summarized in figure 4. Animal reservoir studies showed that O157:H7 is found in animals and animal products in China (Li 2008). In detailed epidemiological studies in Xuzhou city, Jiangsu Province, between 1999 and 2006, Xu and colleagues (Liu *et al.* 2007) reported 131 recorded O157:H7-caused infectious diarrhoea cases, with HUS complications and mortality of 87.79 per cent. The high epidemic season is from June to September and a systematic surveillance system has been established in China led by China CDC and China Animal CDC. Details of recent epidemics, animal reservoirs and bacterial isolation can be found in relevant websites (http://www.chinacdc.net.cn; http://www.epizoo.org/ch/).

Molecular sequence analysis shows that pathogenic *E. coli* contains extra genes as a result of horizontal gene transfer in comparison with non-pathogenic *E. coli* K12 (Blattner *et al.* 1997). EHEC has also some specialized genes encoding, for example, a type III secretion system that secretes virulence factors for attaching and effacing, and shiga toxin. Strategies for the control of EHEC can be directed to targeting these special factors, e.g. neutralization of shiga toxin. We have focused on trying to interfere with EHEC adhesion to the intestinal cell surface and recently proposed a binding model of intimin to the Tir (translocated intimin receptor; both of these two proteins are bacteria-encoded) linking the intestinal cell surface to EHEC (Y. Ma and G. F. Gao 2009, unpublished data). This model can provide the basis for the design of new drug targets to interfere with EHEC infection.

## 6. Porcine reproductive and respiratory syndrome virus and food safety

PRRSV is emerging as one the major infective agents in the pig industry worldwide since its appearance in the 1980s. It was first diagnosed and isolated in the USA in 1987 (Albina 1997), immediately found in Europe (Wensvoort *et al.* 1991), soon spread to the rest of the world (Blaha 2000). The disease is characterized by reproductive failure in pregnant sows and respiratory distress particularly in suckling piglets, thereupon getting its name. PRRSV, together with lactate dehydrogenase-elevating virus of mice, equine arteritis virus and simian haemorrhagic fever virus, is a single-stranded positve RNA virus and a member of the family Arteriviridae in the order of Nidovirales (Cavanagh 1997). Based on phylogenetic analysis of different virus isolates around the world, PRRSV can be differentiated into two genotypes: Type I, represented by the European prototype Lelystad strain LV, and Type II, the prototype being the Northern American ATCC strain VR2332. Extensive molecular studies show that PRRSV is highly variable in virulence, sequence diversity and antigenicity (Stadejek *et al.* 2006; An *et al.* 2007). Chinese isolates are members of the Type II genotype (Gao *et al.* 2004).

**Figure 5.**
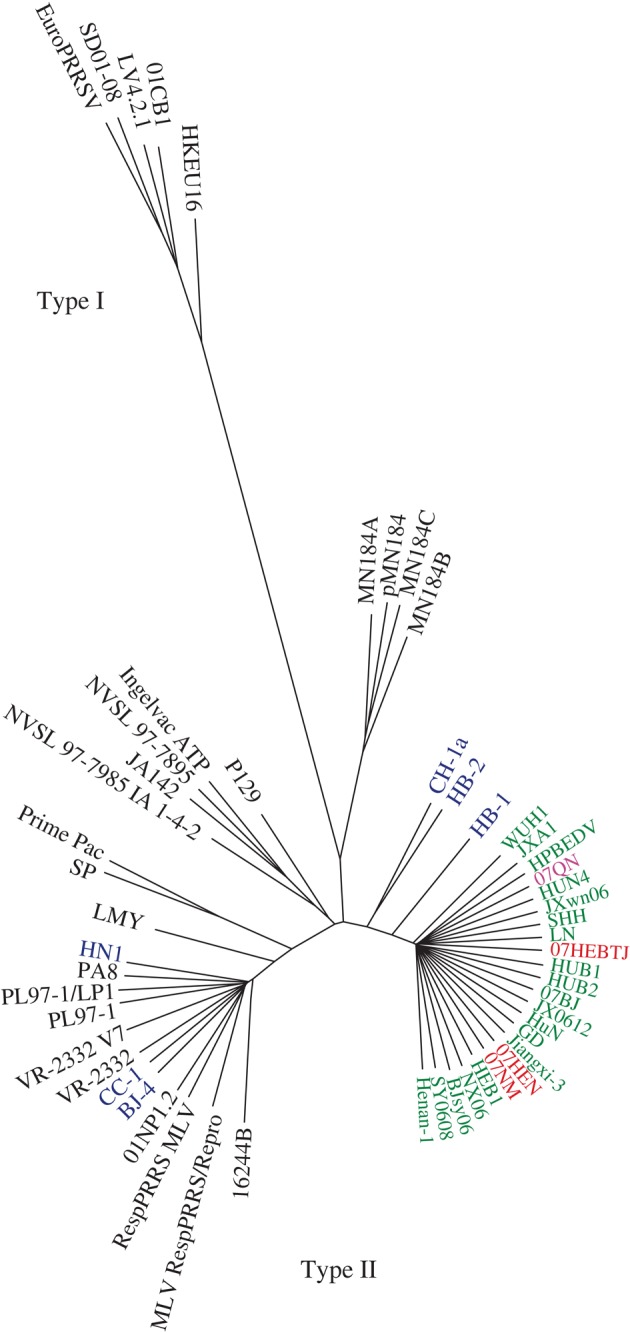
Phylogenetics based on PRRSV whole-genome alignment. All Chinese strains belong to Type II (North American Type). Strains circulated in China before 2006 (blue), in 2006 (green), and in 2007 (red) and in Vietnam in 2007 (pink) are shown.

In 2006, a new type of PRRSV variant was identified in China with high pathogenicity (figure 5), which devastated the pig industry and affected food safety (Tian *et al.* 2007). Parenthetically, from that perspective, an outbreak of PRRSV in the swine population may have important implications for society and human life despite not being a zoonotic human disease. This Chinese variant of PRRSV was found in 2007 in Vietnam where it caused a serious epidemic (Feng *et al.* 2008; figure 5). This variant, which contains a 30-amino acid discontinuous deletion in the non-structural protein NSP2 and some other point mutations in other genomic regions, shows high virulence in both adults (including sows) and piglets. In our studies, the disease was reproduced in experimental infections, fulfilling Koch's postulates (Tian *et al.* 2007). Using reverse genetics, Yuan and colleagues (Lv *et al.* 2008) reproduced the disease from an infectious clone produced from a field isolate JX143, further confirming the high virulence of this epidemic variant of PRRSV.

Variants with NSP2 deletions associated with a high virulence have been found before in the USA but with deletions in different amino acid regions, e.g. isolates MN184A/B/C, P129, etc. Experimental studies with different deletions of the *NSP*2 gene in an infectious clone showed that this gene harbours a virulence factor (Han *et al.* 2007). However, it is still too early to conclude that PRRS viruses causing the devastating 2006–2007 China–Vietnam outbreaks are special in their NSP2 gene being responsible for their high virulence. Further studies are needed in the future to determine whether the NSP2 is definitely a critical feature of virulence.

Since the 2006 outbreaks in China, the pig industries have been seriously affected and food safety is at risk. Pork is an important part of the food chain in China and China consumes more pork than any other country. Great efforts have been made to control this devastating outbreak and new vaccines (both inactivated and attenuated) are under development. Putative receptors for PRRSV have been proposed (Vanderheijden *et al.* 2003; Calvert *et al.* 2007), one of them being CD163 (Calvert *et al.* 2007). CD163s in human and swine are highly conserved and the possible use of human CD163 by PRRSV needs to be pursued in the near future.

## 7. Future perspectives

It seems that many emerging and re-emerging pathogens (mainly virus and bacteria) have zoonotic characteristics and some new human infectious diseases have some animal origins, either as an interspecies cross-infection host, as a vector or as a reservoir host. An urgent call for harmonious relationships between animals, human beings and the environment has rung loudly and a new interdisciplinary research scientific field termed *Eco-Health* has arisen. A new journal addressing this issue by focusing on ecology and health has been established as *EcoHealth* (http://www.ecohealth.net). While human health is our primary goal, we cannot reach this goal without caring for animals and the environment. The surveillance of infectious agents must cover all possible aspects, including animals (wild or domestic) and their eco-environment. The SARS event also has a significant implication: no country can be free in the face of an outbreak of an infectious agent. ‘Someone's sniff in Hong Kong made the ambulance in Toronto run around the city’. A pathogen has no border. We need to work together to tackle emerging and re-emerging pathogens and pathogen surveillance needs to be internationally coordinated, maybe through the establishment of a collaborative network.
